# A Re-Audit of Physical Health Monitoring of Day-Care Patients in the Adult Eating Disorder Service at Surrey and Borders Partnership NHS Foundation Trust

**DOI:** 10.1192/bjo.2024.569

**Published:** 2024-08-01

**Authors:** Amit Fulmali, Tayeem Pathan

**Affiliations:** Surrey and Borders Partnership NHS Foundation Trust, Chertsey, United Kingdom

## Abstract

**Aims:**

To determine if the physical health monitoring of day-care patients in the Adult Eating disorder service (AEDS) is done in line with the recommendations of NICE guidelines and relevant Medical Emergencies in Eating Disorders (MEED) Guidance on Recognition and Management.

**Methods:**

1. For every attendance of patients to the day-care Clinic it is expected that the physical health monitoring to be offered would include:

- Weight
- Height (if first attendance)
- BMI
- HR (Pulse rate)
- Sitting/Standing BP
- Temperature

2. Relevant blood tests and ECGs on a schedule based on patient's BMI or as needed based on clinical indication.

23 patients were identified as having been seen in AEDS day-care centre between April 2021 till the point of discharge. 9 were deemed inappropriate due to incomplete information. Of the remaining 14, 9 patients were randomly selected, their documentation were looked from admission to day-care to the point of discharge. The monitoring was audited at 3 points of contact over the course of their first clinic appointment, the middle and point of discharge.

**Results:**

1. Comparing data from previous audit, the average admission in day-care decreased from 5.5 to 3.5 months.
2. There was overall improvement in the ECG and blood test monitoring.
3. At the admission and the last assessment there was 100% monitoring of BMI, weight, blood pressure and pulse.
4. There was a drop in temperature monitoring by 11.1% in the first and last assessment due to faulty equipment.
5. The ECG and bloods percentage dropped by 11.1% at all the monitoring points.
6. At the midpoint there was no documentation of BMI, Blood Pressure, and pulse for 1 patient.

**Conclusion:**

1. Investigations were delayed from the patient's side.
2. Due to COVID there was difficulty in accessing the primary care appointments for investigations.
3. The temperature equipment was not working properly.

**Recommendations:**

1. Keeping a fixed format for documenting day-care visits on the SystmOne software. A Sample format made available for documentation.
2. Document all the parameters checked in the patients’ electronic records on the same day.
3. Day-care clinical team to upskill on ECG via training.
4. Team Resources to be allocated to have in-house ECG in day-care.
5. SUSS test to be done for all RED (High risk) patients as clinically indicated and clearly document in the notes, e.g. SUSS: done/not done and reason with date SUSS conducted on.

